# Intergenerational Effects of Gamma Radiation on Biology and Transcriptome of Invasive Tomato Leaf Miner, *Tuta absoluta*

**DOI:** 10.3390/insects16101062

**Published:** 2025-10-17

**Authors:** Yuhan Pan, Haixia Zhang, Qinghe Zhang, Farman Ullah, Yiming Pan, Yaru Wang, Limin Chen, Xiaowei Li, Jinming Zhang, Shuxing Zhou, Yaobin Lu, Youming Hou

**Affiliations:** 1 State Key Laboratory of Agricultural and Forestry Biosecurity, Fujian Agriculture and Forestry University, Fuzhou 350002, China; panyuh0306@163.com (Y.P.); zhx0422980802@163.com (H.Z.); wououk@163.com (Q.Z.); 2 State Key Laboratory for Quality and Safety of Agro-Products, Institute of Plant Protection and Microbiology, Zhejiang Academy of Agricultural Sciences, Hangzhou 310021, China; farmanullah787@gmail.com (F.U.); pym9248@163.com (Y.P.); wangyaru0617@163.com (Y.W.); clmit@zju.edu.cn (L.C.); lixiaowei1005@163.com (X.L.); zhanginsect@163.com (J.Z.); 3 Lishui Institute of Agriculture and Forestry Sciences, Lishui 323000, China; 4 Xianghu Laboratory, Institute of Bio-Interaction, Hangzhou 311231, China

**Keywords:** Sterile Insect Technique (SIT), gamma radiation, inherited sterility, gene expression, life table, fecundity

## Abstract

**Simple Summary:**

This study examined how gamma radiation at a substerilizing dose of 300 Gy affects the invasive tomato pest *Tuta absoluta*, focusing on male moths and their F1 offspring. An age-stage, two-sex life table approach was used to assess the F1 generation’s development, survival, and reproduction. Compared to non-irradiated groups, the irradiated F1 moths experienced longer development times, lower survival rates, shorter lifespans, and fewer offspring. Key population metrics also declined, with some even turning negative, indicating population decline may occur. Additionally, transcriptomic analysis identified 232 differentially expressed genes between irradiated and non-irradiated groups. These genes are involved in crucial processes such as metabolism, hormone production, and immune response. The expression levels of 13 key genes related to male fertility were reduced in irradiated males. Overall, this study shows that irradiated male *T. absoluta* can pass harmful effects to their offspring, impairing growth and reproduction. This provides a theoretical basis for controlling *T. absoluta* through the sterile insect technique and highlights potential genetic targets for a genetic sterile insect technique strategy.

**Abstract:**

The tomato leaf miner, *Tuta absoluta*, is a major pest affecting economically important crops like tomatoes, causing significant global economic losses and exhibiting increasing resistance to pesticides. The sterile insect technique (SIT) is an environmentally friendly control method that is sustainable for both ecosystems and human health. This study used age-stage, two-sex life tables, transcriptomics, and bioinformatics to analyze how irradiation affects the reproductive capacity of male *T. absoluta*. Compared to the control group, the irradiated offspring showed reduced total lifespan, pre-adult survival rate, net reproductive rate, and intrinsic growth rate. Transcriptomic analysis identified 232 differentially expressed genes (DEGs). GO and KEGG enrichment analyses revealed that irradiation impacted biological processes in male adults related to key biomolecules, hormone metabolism and synthesis, and immune responses. Of the 14 selected genes validated through RT-qPCR, 13 were identified as potential regulators of male reproductive capacity, offering possible targets for controlling *T. absoluta* using inherited sterility-based SIT strategies. Overall, this study provides a theoretical basis for applying SIT in field control and identifies potential genetic targets for managing *T. absoluta* populations through a genetic sterile insect technique.

## 1. Introduction

The tomato leaf miner, *Tuta absoluta*, also known as the South American tomato moth (Lepidoptera: Gelechiidae), was first identified in 1917 in Huancayo, Peru. By the 1950s, it had become a significant pest impacting the tomato industry in South America [1,2]. From the 1990s onwards, the insect spread and established itself in nearly all South American countries [1]. Currently, *T. absoluta* has successfully invaded over 110 countries and regions worldwide [3,4]. In China, this pest was first detected damaging open-field fresh tomatoes in Xinjiang in August 2017. To date, it has been reported in multiple provinces and municipalities, including Xinjiang, Yunnan, Shaanxi, Ningxia, and Beijing, causing varying degrees of damage to local tomato production and posing a serious threat to the healthy development of China’s tomato industry [5].

Currently, chemical control remains the primary method for managing *T. absoluta* [6], despite its several unintended effects on beneficial insects [7]. However, these chemical insecticides break down to sublethal concentrations through biotic and abiotic factors, which can cause both sublethal/hormetic effects and resistance development [8,9]. The widespread use of insecticides may lead to insecticide resistance in the pest population. Moreover, *T. absoluta* larvae exhibit strong concealment behaviors by mining into host plant tissues such as leaves, axillary buds, tender stems, and immature fruits. This feeding habit creates a natural protective barrier that greatly reduces the effectiveness of insecticides. Therefore, there is an urgent need to develop effective, eco-friendly green management technologies specifically targeting invasive insect pests [10]. The Sterile Insect Technique (SIT) employs ionizing radiation to induce male sterility, followed by sustained mass releases in infested areas. By competing with wild males for mating opportunities with fertile females, this method can significantly reduce or even eradicate target pest populations. As an environmentally safe method, SIT is a vital component of Integrated Pest Management (IPM) strategies. Currently, SIT has been successfully used in controlling and eradicating several major invasive pests, including *Ceratitis capitata*, *Zeugodacus cucurbitae*, and *Cydia pomonella* [11,12]. Notably, this technique substantially provides significant environmental benefits as an eco-friendly and harmless strategy for humans and non-target organisms. These qualities make SIT a promising green solution for managing *T. absoluta* [13].

Previous studies on radiation-induced sterility in lepidopteran pests have shown that females are more sensitive to radiation than males. When treated with substerilizing doses, male moths display increased mating competitiveness while passing sterility to their offspring. Inherited Sterility (IS) is a key strategy for managing lepidopteran pests, with substerilizing doses causing partial sterility in males and full sterility in females. Importantly, the genetic damage caused by radiation is inherited by the F1 generation through chromosomes, resulting in offspring sterility. Recent progress has been made in developing IS techniques for *T. absoluta*. The use of X-ray irradiation at 200 Gy produced a dose-dependent sterilizing effect, causing complete sterility in females and partial sterility in males [14]. Field cage experiments with a 15:1 release ratio (irradiated to non-irradiated moths at a balanced sex ratio) showed that this treatment significantly lowered the number of offspring compared to control groups [15]. Zhou et al. found that gamma-irradiation at 300 Gy causes full female sterility and partial male sterility in *T. absoluta*. This dose maintains normal eclosion rates and mating competitiveness in the irradiated male group while effectively inducing F1 sterility, leading to a notable reduction in offspring numbers [16].

The two-sex life table considers how male individuals influence population dynamics [17]. Using life table analysis is important for further exploring its effectiveness in pest control. Since most pest species show sexual dimorphism and age-stage developmental traits, limited knowledge of their developmental stages can delay pest management [18]. Both male and female adults, as well as larvae of *T. absoluta,* can damage host plants. Using a two-sex life table analysis provides a more complete assessment of this damage, which is crucial for understanding the growth of irradiated offspring and developing more effective control strategies. This leads to the question: which genes are linked to the inherited sterility effects caused by substerilizing doses? To explore this, our study used transcriptome sequencing to compare gene expression differences between F1 generation male adults irradiated at 300 Gy and normal male adults. This approach aims to uncover the molecular mechanisms behind sterility, identify male-specific genes associated with inherited sterility in *T. absoluta*, and suggest potential molecular targets for designing inherited sterility-based SIT strategies against this damaging pest.

## 2. Materials and Methods

### 2.1. Insect Rearing

The *T. absoluta* population used in this study was collected from tomato fields in Yuxi, Yunnan Province, in June 2019. The collected population was maintained in the laboratory on tomato host plant under controlled environmental conditions (25 ± 1 °C, 60–70% RH, 8:16 h L:D) to establish an experimental colony. Throughout the rearing process, the *T. absoluta* population remained unexposed to any environmental stressors.

### 2.2. Irradiation

Pupae of *T. absoluta* were collected 2–3 days before emergence and sexed based on genital positioning (with female and male genital pores located on the 8th and 9th abdominal segments, respectively) [19]. Male pupae were selected as the F0 generation and irradiated with a ^137^Cs-γ radiation source (model xN658, activity 7.77 × 10^14^ Bq, Radiochemistry Center, Buckinghamshire, UK) at a dose of 300 Gy, with a dose rate of 1 Gy/min. Non-irradiated male pupae served as controls. For each irradiation replicate, 5 male pupae were placed in glass tubes (1.5 cm diameter × 8.0 cm height), with ten replicates processed per irradiation session. The irradiated pupae were housed individually housed in 10 mL centrifuge tubes with ventilation holes. Virgin male adults (2 days old) from the F1 generation were collected from irradiated males × normal females (IR group) and non-irradiated males × normal females (CK group). All emerged adults were maintained on 10% honey–water-soaked cotton as a nutritional source. For transcriptome analysis, 30 male adults were collected from each group (CK and IR), with three biological replicates per group.

### 2.3. Life Table Study

Virgin F0 male adults that successfully emerged from irradiated pupae were individually paired with untreated virgin females in glass tubes (3.0 cm diameter × 10.0 cm height) containing fresh tomato leaves. In total, ten irradiated male adults and ten untreated female adults were copulated, and 10% honey–water was supplied to provide supplementary nutrition. Tomato leaves were replaced daily until oviposition ceased, at which point the total egg production per female was recorded. Eggs on each leaf were transferred daily onto fresh tomato leaves in Petri dishes (9.0 cm diameter × 1.5 cm height). From the oviposition clusters, 820 eggs from the treatment group and 320 eggs from the control group were randomly selected for subsequent analysis. The following parameters were documented for the F1 generation: egg hatch rate, larval-to-adult survival rate (from egg to adult emergence), adult sex ratio, and developmental duration at each stage. These metrics were analysed to assess the effect of 300 Gy gamma irradiation on F1 progeny development. Daily records were kept for both male and female adult survival rates and fecundity until mortality. In cases where individual moths died prior to mating, replacements were chosen from the same population cohorts to maintain the experiment. Data from deceased individuals were excluded from life table analysis. For both control (CK) and treatment (IR) groups, F1 female adults were mated with non-irradiated male adults, with oviposition records maintained for each pairing.

### 2.4. Life Table Data Analysis

Raw data of *T. absoluta* were analysed using the TWOSEX-MSChart program (https://www.faas.cn/cms/sitemanage/index.shtml?siteId=810640925913080000) “URL (accessed on 25 July 2025)” [20], incorporating both sexual dimorphism and individual developmental rate variations. The following demographic parameters for *T. absoluta* were calculated: developmental duration, fecundity, mortality rate, net reproductive rate (*R*_0_), intrinsic rate of increase (*r*), finite rate of increase (*λ*), and mean generation time (*T*).

The *l_x_* and *m_x_* were determined using Equations (1) and (2):

(1) $$l_{x}=\sum_{j=1}^{\beta }s_{xj}$$

(2) $$m_{x}=\frac{\sum_{j=1}^{\beta }s_{xj}f_{xj}}{\sum_{j=1}^{k}s_{xj}}$$

where *s_xj_* represents the possibility that a newly born nymph will survive to age *x* and stage *j*. *β* shows number of stages, while *f_xj_* represents age-stage specific fecundity of the individual at age *x* and stage *j*.

The standard errors for developmental duration, female adult pre-oviposition period (APOP), total pre-oviposition period (TPOP), oviposition, fecundity, and life table parameters were estimated using bootstrap resampling [21]. All standard errors were calculated through 100,000 bootstrap iterations using the bootstrap technique implemented in the TWOSEX-MSChart software [22].

### 2.5. RNA Extraction and Transcriptome Sequencing

Total RNA was extracted from 2-day-old F1 male adults (both irradiated and non-irradiated groups) using the Trizol method. RNA purity and concentration were measured with a NanoDrop 2000 spectrophotometer (Thermo, Wilmington, DE, USA), while RNA integrity was assessed using an Agilent 2100 Bioanalyzer/LabChip GX system (PerkinElmer, Waltham, MA, USA). The eukaryotic mRNA was enriched using oligo(dT)-coupled magnetic beads, followed by fragmentation in Fragmentation Buffer. Using the fragmented mRNA as template, first-strand cDNA was synthesized and subsequently converted to double-stranded cDNA. After purification, the double-stranded cDNA underwent end repair, A-tailing, and sequencing adapter ligation. Fragment size selection was performed using AMPure XP beads (Yeasen, Shanghai, China), followed by PCR amplification to construct the final cDNA library. Following library construction, the cDNA library was first quantified using a Qubit 3.0 Fluorometer (Invitrogen, Carlsbad, CA, USA), with concentration requirements exceeding 1 ng/μL. Subsequently, the insert size distribution was verified using the Qsep400 High-throughput Analysis System (Bioptic, Changzhou, China). Upon confirmation of expected insert sizes, the library’s effective concentration (>2 nM) was precisely quantified by RT-qPCR to ensure quality. Finally, qualified libraries were sequenced on a high-throughput sequencing platform in PE150 mode. The clean data were mapped to the reference *Tuta_absoluta* genome version IBG_00819 (http://v2.insect-genome.com/Tabs) “URL (accessed on 20 March 2023)”. The selected gene fragments were subjected to functional enrichment analysis using Gene Ontology (GO) and pathway enrichment analysis through the Kyoto Encyclopedia of Genes and Genomes (KEGG) database.

### 2.6. Quantitative Real-Time PCR

RT-qPCR analysis was performed for the 14 candidate genes using the PrimeScript™ RT Master Mix (Perfect Real Time) cDNA synthesis kit (Takara, Dalian, China) and TB Green™ Premix Ex Taq™ II (Tli RNaseH Plus) (Takara, Dalian, China), with *TaEF1-α* serving as the reference gene. Three biological replicates and three technical replicates were included for each treatment, with primers listed in Table 1.

### 2.7. Statistical Analysis

The experiment included three biological replicates for each treatment group, with each sample analyzed in three technical replicates. Gene relative expression levels were calculated using the 2^−ΔΔCT^ method based on RT-qPCR results. Statistical analysis was performed using SPSS software (version 21.0; IBM, Armonk, NY, USA). The normality of the data was verified using Shapiro–Wilk tests. After confirming the normal distribution, gene expression differences between irradiated and non-irradiated groups were compared using independent samples *t*-tests. Data visualization and curve fitting were conducted using GraphPad Prism statistical software (version 8, GraphPad Software, San Diego, CA, USA, www.graphpad.com).

## 3. Results

### 3.1. Development Time, Survival, and Oviposition Period

Significant differences in egg-to-pupation developmental duration, egg hatch rate, adult pre-oviposition period (APOP) and total pre-oviposition period (TPOP) were analyzed between the control and treatment groups (Table 2). Compared to the control group, the egg hatch rate of offspring produced by irradiated males showed an extremely significant reduction (*p* < 0.001). The egg hatching duration was slightly shortened, showing no statistically significant difference between treatments (*p* = 0.21129). Compared to the F1 generation of the control group, the irradiated F1 larvae showed significantly prolonged developmental durations across all stages (the 1st instar larvae, 2nd instar larvae, 3rd instar larvae, 4th instar larvae, and pupal stage showed *p* < 0.001, *p* < 0.001, *p* = 0.0155, *p* < 0.001, and *p* < 0.001, respectively). The APOP of the F1 generation in the irradiated treatment group was 1.24 days longer than that in the non-irradiated group, with a significant difference (*p* = 0.0219). Moreover, the TPOP of the non-irradiated offspring was significantly lower than that of the irradiated offspring (*p* < 0.001).

The differences in pre-adult duration, pre-emergence survival rate, adult longevity, and total longevity between the offspring without irradiation and those irradiated with 300 Gy are shown in Table 3. Compared to the non-irradiated offspring with a pre-adult developmental duration, the irradiated offspring exhibited a significantly prolonged pre-adult developmental period (*p* < 0.001). The pre-emergence survival rate of the F1 generation showed a dramatic reduction following irradiation treatment (*p* < 0.001). The longevity of both female and male adults in the F1 generation of the irradiation treatment group was significantly shorter than the control group (*p* < 0.001).

**Figure 1:**
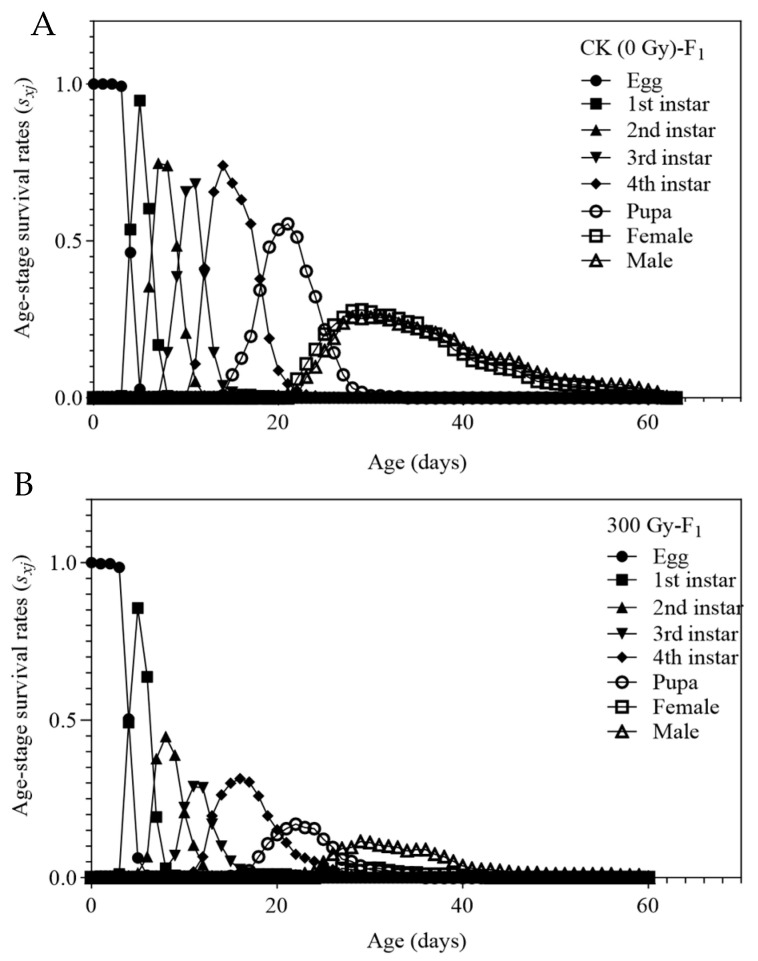
Age-stage -specific survival rate (*s_xj_*) of *Tuta absoluta* F1 generation. (**A**) Survival rate of offspring of male parent treated with no irradiation. (**B**) Survival rate of offspring of male parent treated with 300 Gy gamma irradiation doses.

The survival rates (*s_xj_*) of the F1 generation of *T. absoluta* in both control and 300 Gy irradiated groups are shown in Figure 1. The daily larval survival rate was significantly lower in the irradiated treatment group compared to the control. A shorter *x*-axis span of the *s_xj_* curve indicates a shorter instar duration and faster developmental rate for individual insects. The overlapping *s_xj_* curves at different ages of *T. absoluta* suggest generational overlap, where various developmental stages (instars) occur simultaneously due to individual differences in growth rates. Both control and irradiated groups displayed gradually declining survival rates, but the irradiated treatment group showed significantly lower survival rates during the 2nd, 3rd, and 4th instar larval stages, as well as reduced pupal and adult survival. These findings demonstrate that 300 Gy of gamma radiation markedly inhibits the growth and development of the F1 generation *T. absoluta*.

### 3.2. Life Table Parameters

The population parameters and fecundity of *T. absoluta* calculated using the age-stage, two-sex life table program under different treatments are presented in Table 4. The number of eggs laid per female in the F1 generation after irradiation was only 36.8% of that in the control group, with significant reductions in fecundity (*p* < 0.001). Compared with the control group, both the intrinsic rate of increase (*r*) (*p* < 0.001) and the finite rate of increase (*λ*) (*p* < 0.001) in the 300 Gy of gamma radiation group were extremely significantly lower than those in the control group. The mean generation time (*T*) of the irradiation treatment group was 2.8 days longer than that of the control group, also showing an extremely significant difference (*p* < 0.001).

After 300 Gy of gamma radiation, the ratio of female adults to male adults in the F1 generation (0.25) was significantly lower than that in the control group (1.127). This indicates that 300 Gy of gamma radiation has a greater effect on female individuals in the offspring than on males, which is unfavourable for population growth. *R*_0_ can be calculated as follows: *R_0_* = *Nf* × *N* × *F*, where *F* is the average fecundity [23]. Therefore, the trend in *R*_0_ in the control group and the treatment groups (*p* < 0.001) is similar to that of the average fecundity (*p* < 0.001).

**Figure 2:**
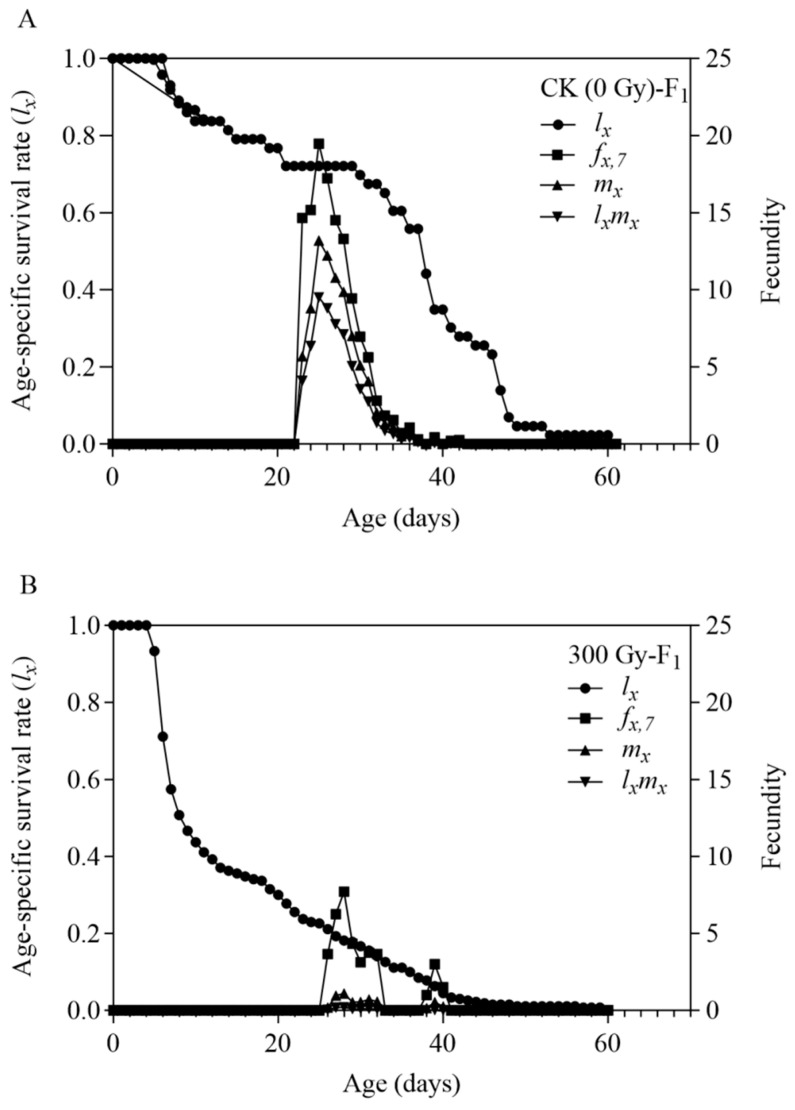
Age-specific survival rate (*l*_x_), age-specific fecundity (*f*_x,7_), age-specific fecundity (*m*_x_) and age-specific net fecundity (*l*_x_*m*_x_) of the F1 population of *Tuta absoluta.* (**A**) Offspring of male parent treated with no irradiation. (**B**) Offspring of male parent treated with 300 Gy gamma irradiation doses.

The age-specific survival rate (*l*_x_) and the age-stage-specific fecundity curve (*f*_x,7_, where 7 indicates the adult stage in *T. absoluta*’s developmental staging system) of the F1 generation are shown in Figure 2. The survival curve (*l_x_*) of the F1 generation in the control group initially fluctuated, then stabilised during mid-development, and gradually declined towards the end. Conversely, the irradiated group displayed an initially stable *l_x_* curve that rapidly declined before slowing down. Combining the developmental duration parameters and the (*s_xj_*) curve suggests that the larval stage and pupal stages of the F1 generation of *T. absoluta* in the 300 Gy of gamma radiation group face a higher risk of mortality. The (*f*_x,7_) curve in the control group shows a rising slope in the early stage and a declining slope falling in the later stage, with minimal fluctuation. In contrast, the (*f*_x,7_) curve of the gamma radiation group fluctuates more irregularly, indicating that the female adults in the F1 generation of the control group have relatively consistent daily fecundity, while those in the gamma radiation group differ in egg production and the egg-laying period.

### 3.3. RNA Quality Control Data Statistics

To further explore the molecular mechanism of inherited sterility in *T. absoluta* and identify its target genes, the transcriptome sequencing was used. Sequencing data are shown in Table 5. After filtering and quality control of the raw data, the CK group produced 19,546,077–26,314,076 clean reads, while the IR group generated 22,573,216–25,163,786 clean reads. Eukaryotic reference-based transcriptome (RNA-seq) analysis of the six samples yielded a total of 41.19 Gb of clean data, with each sample contributing at least 5.82 Gb. The percentage of Q30 bases was 97.70% or higher, and the GC content of the sample sequences ranged from 42.95% to 43.89%. The efficiency of aligning the reads from each sample to the reference genome was between 76.94% and 81.71%. These results demonstrate the high-quality metrics of the transcriptome sequencing data, confirming their suitability for subsequent bioinformatics analyses.

### 3.4. Transcriptome Sequencing Analysis

**Figure 3:**
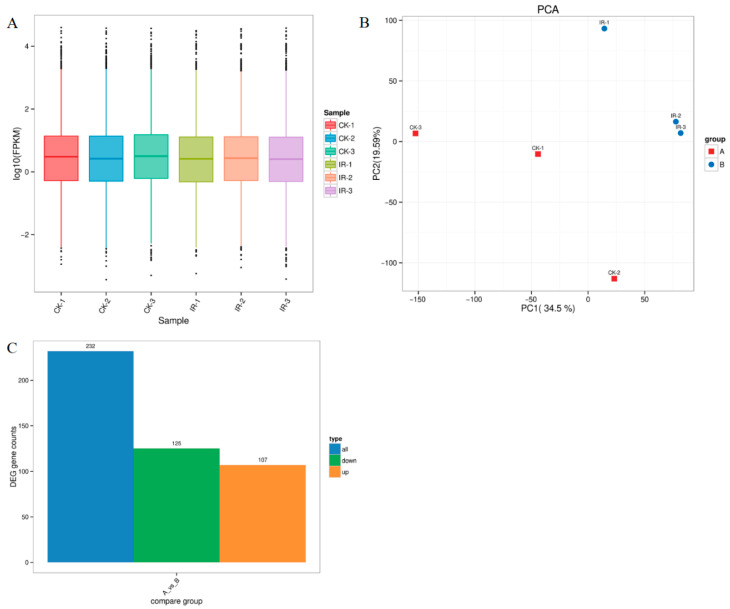
Transcriptome analysis after 300 Gy of gamma radiation. (**A**) Violin plot of gene expression in different treatment groups. (**B**) PCA analysis of the number of expressed samples among different irradiation treatment groups. (**C**) Number of DEGs in different irradiation groups.

The FPKM distribution of genes under different irradiation doses was assessed on a global level. All biological replicates exhibited highly consistent gene expression patterns (Figure 3A), indicating that irradiation treatment did not significantly alter transcriptional profiles. The expression trends demonstrated remarkable reproducibility across all samples. Principal component analysis (PCA) among samples from various treatment groups is shown in Figure 3B. There were notable differences between the two treatment samples. A total of 232 DEGs were detected, with 107 being up-regulated and 125 down-regulated (Figure 3C).

### 3.5. GO Enrichment Analysis

**Figure 4:**
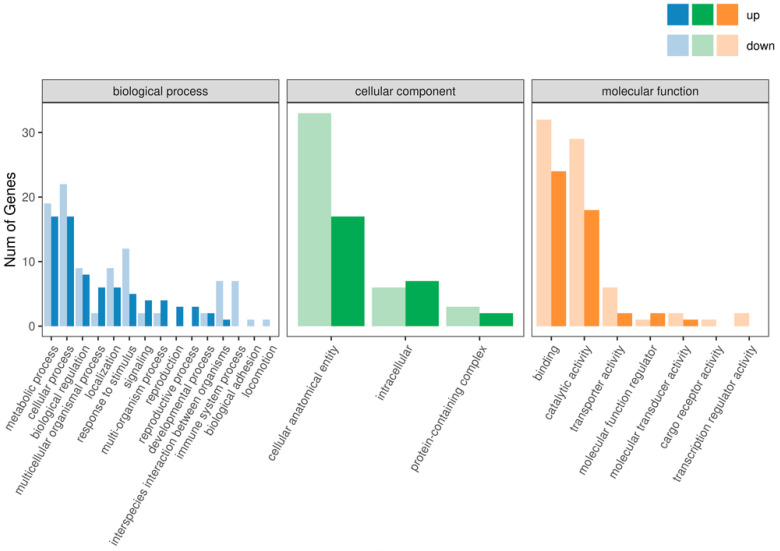
GO function annotation analysis.

In the GO enrichment analysis of the IR vs. CK group, the significantly enriched GO terms with many DEGs are as follows: Cellular Component: integral component of membrane; Molecular Function: nucleic acid binding; Biological Process: biological process; Molecular Function: molecular function; Cellular Component: cellular component; Molecular Function: hydrolase activity; Cellular Component: cytoplasm; Cellular Component: obsolete cell; Molecular Function: metal ion binding; Molecular Function: serine-type endopeptidase activity; Cellular Component: obsolete cell part; and Biological Process: cellular process (Figure 4). These results show the widespread involvement of various biological processes, cellular components, and molecular functions in the development of F1 male offspring from crosses between irradiated *T. absoluta* males and non-irradiated females.

### 3.6. KEGG Pathway Analysis

**Figure 5:**
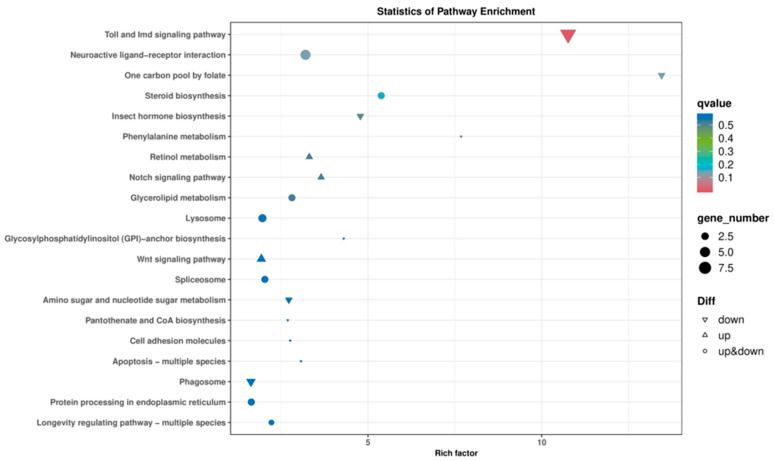
KEGG enrichment analysis.

KEGG analysis was conducted on the DEGs obtained from the IR/CK group, identifying a total of 40 signaling pathways. The top 20 pathways are shown in Figure 5. These DEGs are associated with core metabolic pathways such as glucose metabolism, lipid metabolism, and amino acid metabolism. These findings indicate that, compared to non-irradiated male adults, the anabolic metabolism of essential biological substances in the irradiated F1 generation male adults of *T. absoluta* was impacted.

### 3.7. Identification and Screening of Differentially Expressed Genes (DEGs) Associated with Sterility

**Figure 6:**
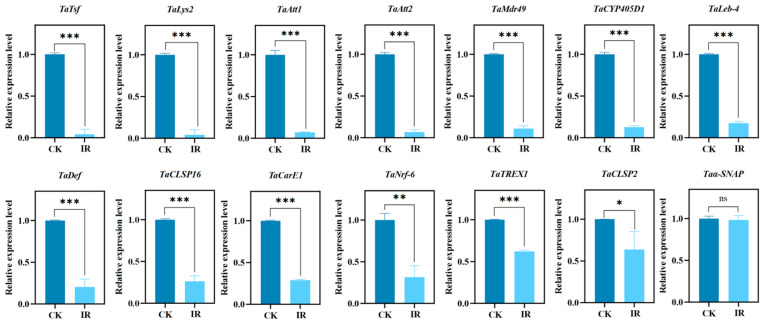
Quantitative verification of key genes of *Tuta absoluta* related to male fertility by RT-qPCR. Data are shown as mean ± SE. Statistical significance was analyzed by independent-samples *t*-test. ns: no significance, (*p* > 0.05), * *p* < 0.05, ** *p* < 0.01, *** *p* < 0.001.

Based on the results above, 14 target genes potentially related to male fertility were identified. *TaTREX1* is involved in replication, recombination, and repair processes. *TaCarE1* functions in carbohydrate transport and metabolism. *TaCYP405D1* and *TaMdr49* are connected to the biosynthesis, transport, and breakdown of secondary metabolites, as well as lipid transport and metabolism. *TaNrf-6* belongs to the acyltransferase family and participates in lipid metabolism. Trypsins *TaCLSP16* and *TaCLSP2*, along with *TaAtt1*, *TaAtt2*, and *TaLys2*, are linked to post-translational modification, protein turnover, and chaperoning. *Taα-SNAP* is involved in intracellular transport, secretion, and vesicle trafficking. Transferrin *TaTsf* relates to energy production and conversion. *TaDef* plays a role in signal transduction. *TaLeb-4* is an antimicrobial peptide key to host defence. RT-qPCR validated the expression of these genes. The findings (Figure 6) indicated that 13 genes were significantly downregulated in the irradiated progeny. These genes will be further studied to explore their association with decreased male fertility in *T. absoluta*.

## 4. Discussion

In this study, by comparing the differences in development, survival, reproduction, and gene expression between the F1 generation of *T. absoluta* irradiated with a sub-sterile dose (300 Gy) of gamma rays and the non-irradiated F1 generation, we systematically evaluated the biological and transcriptomic effects of this irradiation dose on the F1 generation of *T. absoluta*. The results showed that the detrimental effects caused by irradiation can be inherited by the F1 generation, affecting their growth, development, reproduction, and the expression of related genes.

The F1 generation after irradiation treatment showed significant delays in egg hatching and developmental stages of each larval instar, pupal stage, and pre-adult period, along with a substantial decrease in pre-emergence survival rate. This effect has also been seen in other lepidopteran pests such as *Spodoptera frugiperda* and *Ephestia elutella* [24,25]. It may be related to irradiation-induced DNA damage and the suppression of cell division [26,27]. Previous studies suggest that ionizing radiation can cause apoptosis either by directly damaging DNA or by generating reactive oxygen species (ROS) [28,29,30,31], with increased sensitivity during the rapidly developing larval stage. Additionally, the decrease in pupal survival might result from irradiation disrupting hormone synthesis pathways involved in metamorphosis, like juvenile hormone and ecdysone [32,33], which is supported by the finding that the “Insect hormone biosynthesis” pathway was significantly enriched in KEGG analysis.

The number of eggs laid per female in the F1 generation after irradiation was only 36.8% of that in the control group, with extremely significant reductions in both the intrinsic rate of increase and the finite rate of increase. A combination of factors may cause this reproductive inhibition. The female-to-male ratio in the irradiated group was significantly lower than that in the control group, which aligns with the results of Zhou [16], indicating that irradiation exerts stronger selection pressure on the development or survival of female individuals. A similar phenomenon has also been observed in other lepidopteran pests such as *S. frugiperda* [25]. Lepidopteran insects have a WZ/ZZ (female/male) sex determination system [34], and irradiation-induced lethal mutations occur on the Z sex chromosome in the current generation, thereby causing the death of female offspring [35]. In the transcriptome analysis, it was found that 125 genes were down-regulated in the irradiated group. Among them, genes involved in the “Steroid biosynthesis” and “Retinol metabolism” pathways may affect the synthesis of sex hormones [33,36]. Additionally, the enrichment of the Toll and Imd signalling pathway suggests that immune deficiency may further reduce the lifespan and fecundity of adults [37,38]. Another possible reason for the developmental delay and decreased survival rate of the F1 generation after irradiation is that energy is prioritized for maintaining survival rather than reproduction [39,40], which manifests as a significant reduction in the lifespan and fecundity of female adults.

GO analysis showed that the differentially expressed genes were mainly involved in metabolic processes and binding functions, with notable enrichment of “One carbon pool by folate” and “Pantothenate and CoA biosynthesis” in KEGG pathways. Folate metabolism is a key pathway for DNA synthesis and methylation, and its disruption can directly lead to abnormal embryonic development, such as a lower egg hatching rate. Moreover, folate is crucial for germ cell proliferation [41]. Coenzyme A participates in energy metabolism and lipid synthesis [42], which may affect energy storage during the pupal stage and influence adult fecundity.

Furthermore, among the 13 down-regulated genes verified by RT-qPCR, several may be linked to insect sperm function [43]. Some genes are related to sperm production, transfer and storage. Chymotrypsin-like serine proteases (CLSPs) have been reported to play a crucial role in sperm migration and sperm–egg interaction to regulate male fertility [44]. Transferrin (Tsf), with abundant expressed in the testis at sexual maturity stages, may be crucial for spermatogenesis and sperm formation in *Bactrocera dorsalis* [45]. Knocking down the transferrin gene (Tsf) in *Nilaparvata lugens* (brown planthopper) significantly affects its fecundity, hatching rate, and survival rate, and this gene may also be involved in molting [46]. *SeTsf1* in *Spodoptera exigua* (beet armyworm) plays a dual role in regulating antimicrobial immunity and reproductive processes through tissue-specific iron redistribution [47]. Both *NSF* and *αSNAP* play essential roles in cellular transport and fusion; the absence of these proteins can lead to apoptosis [48]. *α-SNAP*/*NSF* serves a key function in the acrosome reaction, a process essential for fertilization [49]. The carboxylesterase *EST 6* modulated male reproductive functions, which may be consequences of its action on sperm transfer, storage and utilization [50]. *TaCYP405D1* is significantly upregulated in tetraniliprole-resistant populations, which is associated with resistance-related fitness costs [51]. Cytochrome P450 (CYP) was found to be associated with sperm morphology and sperm function [52]. The knockout of *MRP9* and *MRP5* resulted in abonormal sperm mitochondria and decreased fertilization rates [53]. *Mdr49* also regulates reproductive functions in *Aedes aegypti* [54]. We speculate that these genes may serve as core factors that ensure male fertility by directly regulating spermatogenesis.

Nevertheless, for successful sperm delivery and fertilization to occur, it is imperative for sperm to overcome challenges present in the external environment, most notably the host immune system [55]. Several genes have been reported to be involved in the immune system. The queen of *Apis mellifera ligustica* (Western honeybee) can store sperm in her specialized sperm storage organ for many years. Lysozyme, a conserved immune effector, has been found to be associated with lower fertility rates, which correlates with higher levels of the immune effector lysozyme, consistent with a trade-off between immunity and fertility [56]. *Wolbachia*, a symbiotic bacterium widely present in insects, can manipulate host reproduction and alter their immune systems [54]. After infecting *Bombyx mori* (silkworm) with two *Wolbachia* strains, wKue and wCauB, and treating them with PGN, analysis of four antimicrobial peptide (AMP) genes—cecropin B, defensin B, attacin, and lebocin 3—showed that *Wolbachia* infection enhances the host’s ability to produce AMPs in response to PGN immune stimulation [57]. The main function of *TREX1* is to degrade cytoplasmic DNA and prevent the activation of innate immune responses, playing an important immune role in many mammals [58]. The rupture of the micronuclear membrane, which exposes DNA contents to nucleases, is a possible mechanism for DNA degradation [59]. We speculate that a similar function may exist in insects, which requires further exploration. The resource competition between immunity and reproduction highlights a fundamental trade-off within organisms [60]. Therefore, these immune-related genes may impact fertility through resource allocation.

Every physiological process, including spermatogenesis and immune responses, rests ultimately on a sustained energy supply [61]. According to life history theory, a trade-off happens when two or more traits compete for the same resources, requiring organisms to balance energy-intensive traits such as reproduction, growth, and longevity [62]. The decrease in longevity and fecundity of the irradiated progeny of *T. absoluta* may result from an energy trade-off between adaptability and life history traits under stress from treatment [63]. In humans and *Drosophila melanogaster*, upregulation of *TORC1* can enhance the oxidative stress response under stress conditions, which is controlled by *cnc*, a transcription factor homologous to the human *Nrf* gene [64]. Ubiquitinated proteins and damaged mitochondria activate *cnc/nrf-2* via p62, leading to the activation of oxidative response genes with the support of *TORC1* upregulation [65,66]. The downregulation of these genes may further explain the decrease in fertility of the irradiated progeny. Thus, we speculate that these genes may modulate the development and reproduction by energy metabolism.

However, all experiments were carried out under laboratory conditions focusing on a single population and radiation dose, which is a limitation of our study. Different populations may vary in radiosensitivity, and radiation dose is closely related to sterility level and competitiveness [67]. Therefore, to apply the sterile insect technique under real field conditions, further research involving the effect of different populations and dose ranges, the field performance of sterile males, and the effectiveness under a semi-field setting is essential. In addition, very small sample sizes were used for the APOP and TPOP parameters in the irradiated group due to the extremely high mortality of its F1 generation. More biological replications need to be performed to confirm this finding.

For the direct application of the sterile insect technique (SIT), although laboratory experiments are essential for isolating the effects of radiation, the behavior and interactions of irradiated progeny under semi-field or field conditions, with factors like predator presence, temperature fluctuations, and host plant quality variation, can lead to divergent outcomes. At the molecular level, transcriptomic studies have identified a strong association between the dysregulation of specific genes and reduced fertility. However, confirming a direct causal link requires future research using gene-editing tools such as CRISPR/Cas9 or RNAi to functionally clarify the roles of these candidate genes. In addition, the current study lacks tissue-level examination, particularly regarding reproductive tissues such as the testis. The effects of irradiation on the morphology and gene expression of reproductive organs will be further explored.

## 5. Conclusions

In summary, exposing male *T. absoluta* to a substerilizing dose (300 Gy) can pass on adverse effects to their offspring. It may impede the offspring’s growth, development, and reproduction by altering the expression of key genes and associated signaling pathways, thereby effectively reducing offspring population growth. This study provides a theoretical basis for applying the sterile insect technique (SIT) in field control and identifies potential targets for preventing and managing *T. absoluta* through the genetic sterile insect technique strategy.

## Figures and Tables

**Table 1:** Primer sequences for RT-qPCR of the 14 target genes.

Gene Name	Forward Primer (5′-3′)	Reverse Primer (5′-3′)
*TaTREX1*	CCGGAGGAAGAAAACAATCA	TGGTCAACCCTGAGATTTCC
*TaCarE1*	ACTCCGAGTGCTGAGGACAT	TCGAGCCAAGCTCCAAGTAT
*TaCYP405D1*	GGTGCTCCTTTCAGAACCAG	TGCACATTCGCTGAGAGTTT
*TaNrf-6*	TACTGATGATGGCGTTCTGC	AAGGTAGACGGCGTACGAGA
*TaCLPS16*	TGTGTGGAGCCTCACTTCTG	CGTTGGACAAAATGACGTTG
*TaCLPS2*	GCAGATGAAGGCAGAAGAAGA	AACCAGCCAAGTAAGGGTAAG
*Taα-SNAP*	CAACACGCCCTTGAGAACTA	TTCCTCATTCTGCTCCTCTAAAC
*TaTsf*	GGAGTTCCGTTACGAAGCAG	AGAGAAGCTGGGATCGTTCA
*TaMdr49*	TGTGTGTGGAGTGGTGACCT	CTTCCAGTAGCTCCCTGCAC
*TaAtt2*	TCATCACCATCCGAACTTCA	TATGATGCGGTGTCACCATT
*TaLeb-4*	CCAAGCGCTAAGAGTTACGG	CGGATCATAAGGCTTCGGTA
*TaDef*	CGTTCTCGTTGTCATGATGG	GATACAGTGCAGTGCGCAAG
*TaLys2*	GACAAGATAAGCCCGGTCAA	AATCCGTGACGCTTGAAGAT
*TaAtt1*	GCATCCCACATACCGACTCT	GAAGGCTGGCATAGTCTTGG
*TaEF1-α*	AGTCTCCTCATACATCAAGAAG	CCTCCTTACGCTCAACAG

**Table 2:** Effects of 300 Gy irradiation doses administered to male parent on their offspring fitness parameters in *Tuta absoluta*.

Stage	*n*	0 Gy	*n*	300 Gy
Egg hatch rate (%)	320	0.922 ± 0.015 a	820	0.331 ± 0.016 b
Egg (day)	285	4.484 ± 0.034 a	270	4.552 ± 0.042 a
First instar (d)	264	2.295 ± 0.040 b	140	2.700 ± 0.057 a
Second instar (d)	236	2.809 ± 0.048 b	108	3.250 ± 0.104 a
Third instar (d)	222	2.991 ± 0.050 b	91	3.220 ± 0.081 a
Fourth instar (d)	196	5.842 ± 0.104 b	77	7.377 ± 0.245 a
Pupa (d)	168	6.690 ± 0.081 b	50	7.280 ± 0.120 a
APOP (d)	25	0.760 ± 0.202 b	3	2.000 ± 0.538 a
TPOP (d)	25	24.560 ± 0.337 b	3	27.667 ± 1.120 a

Note: Means ± SE in the same line followed by the same lowercase letters represent no significant differences between treatments were irradiated using a paired bootstrap test (*p* > 0.05). Abbreviations: 300 Gy, the parents were 300 Gy irradiated male and non-irradiated female adults.

**Table 3:** Effects of 300 Gy of gamma radiation administered to male parents on the offspring longevity and survival in *Tuta absoluta*.

Biological Traits	*n*	0 Gy	*n*	300 Gy
Preadult duration (d)	168	24.940 ± 0.177 b	50	27.300 ± 0.445 a
Preadult survival (s_a_) (%)	285	58.947 ± 2.912 a	270	18.519 ± 2.351 b
Female adult duration (d)	89	16.213 ± 0.884 a	10	7.7 ± 1.771 b
Male adult duration (d)	79	18.734 ± 1.081 a	40	11.075 ± 1.075 b
Total longevity (d)	285	30.744 ± 0.951 a	270	15.619 ± 0.758 b

Note: Means ± SE in the same line followed by the same lowercase letters represent no significant differences between treatments using a paired bootstrap test (*p* > 0.05). Abbreviations: 300 Gy, the parents were 300 Gy irradiated male and non-irradiated female adults.

**Table 4:** Life table parameters showing the effects of 300 Gy of gamma ray-irradiated male parent on their offspring in *Tuta absoluta*.

Demographic Parameters	0 Gy	300 Gy
Fecundity (egg/♀)	146.367 ± 10.782 a	53.847 ± 25.149 b
Intrinsic rate of increase (*r*) (D^−1^)	0.082 ± 0.008 a	−0.005 ± 0.020 b
Finite rate of increase (*λ*) (D^−1^)	1.086 ± 0.009 a	0.995 ± 0.020 b
Net reproduction rate (*R*_0_) (offspring)	9.796 ± 2.096 a	0.863 ± 0.500 b
*Nf* to *Nm* ratio	1.127 ± 0.180 a	0.250 ± 0.091 b
Mean generation time (*T*) (days)	27.729 ± 0.373 b	30.512 ± 0.714 a

Note: Means ± SE in the same line followed by the same lowercase letters represent no significant differences between treatments using a paired bootstrap test (*p* > 0.05). Abbreviations: 300 Gy, the parents were 300 Gy irradiated male and non-irradiated female adults.

**Table 5:** Summary of Illumina RNA-sequencing data.

Samples	Clean Reads	Clean Bases	Q30 (%)	GC Content (%)
CK-1	26,314,076	7,821,653,579	98.52	43.33
CK-2	21,055,084	6,268,920,113	98.69	43.80
CK-3	19,546,077	5,815,720,809	98.57	42.95
IR-1	25,163,786	7,483,281,060	98.64	43.20
IR-2	22,573,216	6,718,516,137	98.56	43.33
IR-3	23,824,739	7,081,532,747	97.70	43.89

## Data Availability

The original contributions presented in this study are included in the article. Further inquiries can be directed to the corresponding authors.
